# Psychological problems and mental disorders in female victims of domestic violence

**DOI:** 10.1192/j.eurpsy.2025.2433

**Published:** 2025-08-26

**Authors:** N. Semenova, M. Kachaeva, K. Tukhtaeva, N. Skibina, L. Nazarova

**Affiliations:** 1 Moscow Research Institute of Psychiatry; 2 V. Serbsky National Medical Research Centre for Psychiatry and Narcology; 3 I.M. Sechenov First Moscow State Medical University, Moscow, Russian Federation

## Abstract

**Introduction:**

Domestic violence against women has increasingly been recognized nationally and internationally as a serious problem. Violence against women is a troubling phenomenon in Russia. Meanwhile, domestic abuse against women often results in severe psychological and mental health problems, including but not limited to depression, anxiety, and post-traumatic stress disorder.

**Objectives:**

The main aim of the study was to find out the psychological and psychiatric consequences of intrafamily violence against women.

**Methods:**

A cohort of 18 females turned to psychologists and psychiatrists and was examined. Details of background, as well as psychological and mental problems, were extracted. Each item was assessed with the help of descriptive statistics.

**Results:**

Research has been carried out on the basis of psychological and psychiatric assessment of women who had a long history of violence by their husbands or partners, including sexual violence and maltreatment. Clinical assessment has revealed depression, anxiety, and adaptation disorders, accompanied by acute and chronic somatic diseases, low self-esteem, post-traumatic stress disorder, and drug abuse.

**Conclusions:**

The research shows the high social and clinical significance of the consequences of abuse against women and also the necessity of domestic violence prevention by legal provisions and multidisciplinary research with the participation of psychiatrists, psychologists, and specialists in the fields of primary healthcare, education, and social protection.

**Disclosure of Interest:**

None Declared

